# A Singular Attempt at Suicide in a Lunatic

**Published:** 1845-07-01

**Authors:** G. N. Burwell


### A Singular Attempt at Suicide in a Lunatic.

Communicated for the Buffalo Medical Journal, by G. N. BURWELL, M. D.

In February, 1843, a German was brought to the Lunatic Asylum attached to the Philadelphia Hospital, Blockley. He had been in this country a number of years, spoke English very intelligibly, and as an occupation, followed the putting up of anatomical preparations and skeletons, becoming thus pretty well versed in anatomy. In this capacity he was well known to most of the prominent physicians and surgeons of Philadelphia.

He had not been insane long; was then subject to violent paroxysms of rage, during which he had to be very carefully watched to prevent him from injuring those about him. A tendency to injure himself had also been noticed at these times, but not in any great degree. During the intervals of the paroxysms he was allowed the use of the halls, etc. as he pleased. He would then be very quiet, although not fully rational.

It was during one of these intervals of quiet that I saw him at one of my regular evening visits to the wards. He came up to me, shook hands, and conversed readily and pleasantly. I remember noticing, in passing the door of his room or cell, three or four crackers lying on a small stand at the head of his bed, which. at his request, had been given him by the nurse.

In about fifteen minutes after leaving him, and while engaged in another ward near by, I received word to come to him in haste, for he was dying. All the nurse could tell me was, that he was heard to fall in his cell, and on going to him, he was found on the floor insensible. He was laid on his bed, and word sent to me. On reaching his bed-side, a glance was sufficient to show that all the difficulty lay in or about his windpipe.

There were violent efforts at respiration, *no cough*, face getting more and more livid every instant, the veins of his neck and temples largely swollen, and his mouth tightly closed. He was then and continued for sometime, perfectly insensible, and while in this state, remained passive in our hands.

I immediately thrust my finger into his mouth, and found it full of mashed cracker; that about the cheeks and teeth was very finely divided and doughy from the mixture of saliva; farther in it was coarser and drier; back in the fauces there were some pieces nearly as large as the end of a man's thumb, while back and below this mass of cracker, and lying directly over the rima-glottidis was a piece of paper folded to four or five thicknesses, and about an inch and a half square. This had evidently been first pushed back into the fauces, and the cracker then swallowed upon it, forcing it down upon the rima-glottidis. He must have done this without breathing, and within half of a minute.

Then came the insensibility and the fall which gave the alarm. For a few minutes it became uncertain whether he would live until I could free his mouth and throat, and as a precaution, instruments were obtained, that if necessary I might open his trachea.

The air began to pass into the larynx before the fauces were entirely cleared out, and immediately he began to manifest some sensibility, showing itself in general efforts to rid himself of us, and in rigidly closing his mouth so that pine sticks had to be obtained to put between his teeth, to enable me to finish clearing out his throat; fits of coughing soon came on, which much assisted me in this. The first words he spoke were violent oaths, deprecating our efforts in saving him, and his passion and anger were excessive for a while afterwards, and very painful to witness.

No injury resulted to him from the attempt.

